# Recent advances on organic blue thermally activated delayed fluorescence (TADF) emitters for organic light-emitting diodes (OLEDs)

**DOI:** 10.3762/bjoc.14.18

**Published:** 2018-01-30

**Authors:** Thanh-Tuân Bui, Fabrice Goubard, Malika Ibrahim-Ouali, Didier Gigmes, Frédéric Dumur

**Affiliations:** 1 Laboratoire de Physicochimie des Polymères et des Interfaces (LPPI), Université de Cergy-Pontoise, 5 mail Gay Lussac, Neuville sur Oise, 95031 Cergy-Pontoise Cedex, France; 2 Aix Marseille Univ, CNRS, Centrale Marseille, iSm2, Marseille, France; 3 Aix Marseille Univ, CNRS, Institut de Chimie Radicalaire ICR, UMR 7273, F-13397 Marseille, France

**Keywords:** blue, electroluminescence, emitter, OLED, TADF

## Abstract

The design of highly emissive and stable blue emitters for organic light emitting diodes (OLEDs) is still a challenge, justifying the intense research activity of the scientific community in this field. Recently, a great deal of interest has been devoted to the elaboration of emitters exhibiting a thermally activated delayed fluorescence (TADF). By a specific molecular design consisting into a minimal overlap between the highest occupied molecular orbital (HOMO) and the lowest unoccupied molecular orbital (LUMO) due to a spatial separation of the electron-donating and the electron-releasing parts, luminescent materials exhibiting small S_1_–T_1_ energy splitting could be obtained, enabling to thermally upconvert the electrons from the triplet to the singlet excited states by reverse intersystem crossing (RISC). By harvesting both singlet and triplet excitons for light emission, OLEDs competing and sometimes overcoming the performance of phosphorescence-based OLEDs could be fabricated, justifying the interest for this new family of materials massively popularized by Chihaya Adachi since 2012. In this review, we proposed to focus on the recent advances in the molecular design of blue TADF emitters for OLEDs during the last few years.

## Introduction

**Figure 1:**
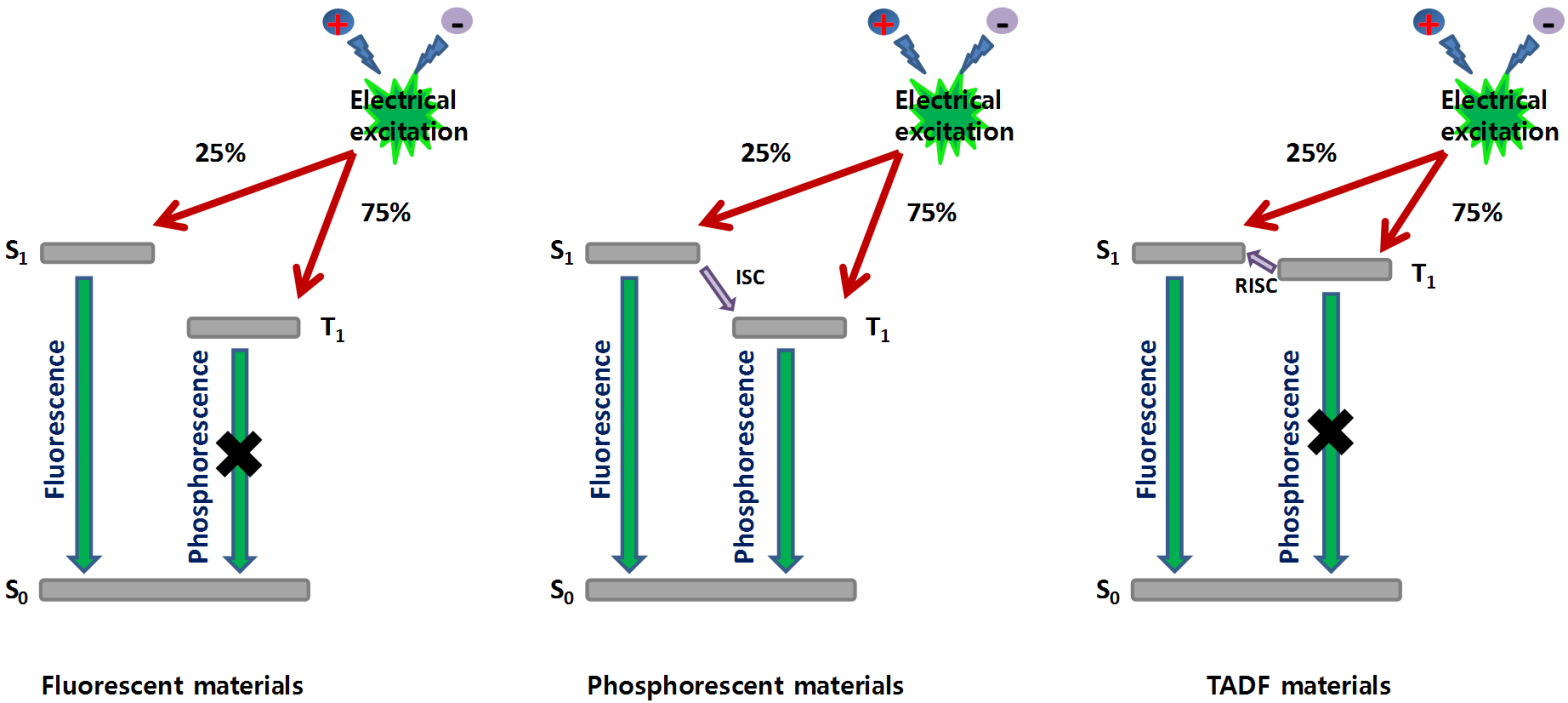
Radiative deactivation pathways existing in fluorescent, phosphorescent and TADF materials.

Since the pioneering works of Tang and VanSlyke in 1987 [1], organic light emitting diodes (OLEDs) have known major evolutions of their structures, not only of the device stacking but also of the materials composing the different layers [2]. The interest of both the scientific and industrial communities for organic electroluminescent devices is supported by the fact that OLEDs have been identified as the key-elements for the fabrication of the next generation display and lighting technology [3]. Notably, lightweight and thin devices can be fabricated onto flexible substrates, favouring the penetration of OLEDs in these two markets. With the aim at reducing the global energy demand on Earth, two parameters govern the power consumption of OLEDs, namely the quantum yield of luminescence of the light emitting material and the device stacking. Indeed, the driving voltage of OLEDs is highly sensitive to the thickness of the different layers, the charge transport ability of the materials but also to their energy levels. By minimizing the energy gaps between adjacent layers and facilitating charge injection from the electrodes, the injection and transportation of holes and electrons can be realized at lower operating voltages. The second parameter concerns the light-emitting ability of the emitter, which is directly related to the nature, and the photoluminescence quantum yield (PLQY) of the emitter. Based on spin statistics, upon electrical excitation, singlet and triplet excitons are formed in a 1:3 ratio [4]. In the case of fluorescent materials, only singlet excitons can be utilized for light emission, limiting the internal quantum efficiency (IQE) of fluorescent OLEDs to 25%. Conversely, phosphorescent materials can both harvest singlet and triplet excitons for emission by intersystem crossing (ISC), enabling to reach a theoretical IQE of 100% for phosphorescent OLEDs [5]. As drawback, triplet emitters are transition-metal complexes mostly based on iridium, platinum and osmium and the scarcity of these metals on Earth, their toxicity and high cost make these materials unsuitable candidates for a mass-production of OLEDs [6]. However, efforts have also been carried out to incorporate emitters comprising less toxic metals, providing mitigate results when tested in devices [7–8]. In 2012, a breakthrough has been obtained by the Adachi group who developed purely organic materials capable to harvest both singlet and triplet excitons for emission [9]. This new family of light emitting materials capable to compete with the well-established triplet emitters and displaying a similar efficiency in devices by developing a new emission mechanism was immediately termed as the third generation of OLEDs emitters that consists of thermally activated delayed fluorescence (TADF) emitters. As specificity, these materials can thermally repopulate the singlet state from the triplet state by reverse intersystem crossing (RISC), leading to an increase of the luminescence intensity. From the OLEDs viewpoint, TADF emitters behave by harvesting both singlet and triplet excitons for radiative transition, excepted that the emission occurs from the singlet state and not from the triplet state (as observed for metal complexes) and that the triplet–triplet annihilation commonly observed with phosphorescent OLEDs [10] can be drastically reduced (see Figure 1). TADF materials can also be metal-free, addressing the fabrication cost and environmental issues. Therefore, TADF emitters retain the high efficiency of the second generation of emitters (triplet emitters), the stability of the first generation of fluorescent materials while eliminating the different problems observed with the two previous generations: triplet–triplet annihilation and low device stability for phosphorescent OLEDs, low IQE for fluorescent OLEDs.

To get full-color displays or white-light OLEDs, the combination of the three primary colors red green blue (RGB) is indispensable. At present, highly emissive and stable blue emitters are actively researched [11–16]. Several points justify the low availability of highly efficient blue materials. First, due to their large bandgaps (Δ*E* > 3 eV), charge injection from the adjacent layers is often difficult, requiring devices to be operated at high voltages [17]. Second, and still related to their wide bandgaps, the probability to transfer an electron from the ground to the excited stable state is considerably reduced, providing materials displaying PLQY greatly reduced compared to that observed for the other colors [18–19]. To end, the propensity of blue emitters to rapidly degradate upon device operation is well established, resulting in a fast and irreversible color shift [20–21]. In this context, TADF blue emitters have been identified as promising candidates to address the color purity, quantum efficiency and long-term device stability issues. Due to the enthusiasm of the scientific community for TADF emitters, this research field evolves extremely rapidly. In this review, a summary of the strategies developed during the last years to design organic blue TADF emitters is presented. It has to be noticed that the values of EQEs reported in the different tables correspond to the maximum EQEs, because many articles do not give sufficient data concerning EQE at the practically relevant luminance of 100 cd/m^2^.

## Review

### 1. Molecular design rules to produce a delayed fluorescence

The efficiency of OLEDs is intimately related to the ability of the light-emitting materials to convert a maximum of injected charges into photons. To optimize this, the TADF process is the most promising strategy as it allows converting the generated and lost triplet excitons of the classical fluorescent materials into emissive singlets. By efficiently upconverting the triplet excitons from the triplet (T_1_) to the singlet state (S_1_), the intrinsic limitation of 25% imposed to fluorescent materials by the 1:3 singlet–triplet ratio can be overcome and an ultimate IQE of 100% can be realized with TADF materials. To promote the endothermic RISC, the energy gap between S_1_ and T_1_ (Δ*E*_ST_) plays a key role and should be as small as possible. From a molecular design viewpoint, Δ*E*_ST_ can be drastically reduced if the highest occupied molecular orbital (HOMO) and the lowest unoccupied molecular orbital (LUMO) are spatially separated, what can be obtained by a suitable steric hindrance that introduces an internal twist and interrupts the π-conjugation but also by a sufficient distance between the electron-donating and the electron-accepting moieties [22–25]. In the design of TADF materials, it should be mentioned the major importance of the spin–orbit vibronic coupling, in addition to the small Δ*E*_ST_. Indeed, a small Δ*E*_ST_ is not sufficient to ensure for a TADF material an efficient RISC which is a vibronically coupled, spin–orbit coupling process with the involvement of the charge transfer state. To remain efficient, the spin–orbit coupling should still have a significant value, even if the separation of the HOMO and LUMO wavefunctions remain a requirement to minimize Δ*E*_ST_. At present, systematic investigations examining the correlation between the spin–orbit coupling and RISC are still scarce [26–29]. Considering that the singlet–triplet energy splitting is one of the key elements for controlling the RISC efficiency, that the dihedral angle between the donor and the acceptor can be difficultly anticipated and that an overlap of both the HOMO/LUMO energy levels could adversely affect the color purity and Δ*E*_ST_, it has to be noticed that the photophysical properties and the geometry of molecules that are suspected to be TADF emitters are often investigated by theoretical calculations prior to synthesis, optimizing the chance to get suitable energy levels and the desired Δ*E*_ST_. This strategy was notably applied to the design of TADF blue emitters containing triarylboron accepting units. Besides, as we will see in this review, the design of a good TADF material by optimizing its structure by theoretical calculations is not sufficient to ensure the fabrication of highly emissive OLEDs. As observed for phosphorescent emitters, optimization of the device stacking, an appropriate choice of the host as well as the materials in the adjacent layers, an adequate dopant concentration, and the efficient confinement of excitons within the emissive layer are primordial parameters to elaborate high performance OLEDs while maintaining the color purity [30]. Due to the difficulty to address simultaneously these different points, numerous light emitting materials have been revisited several times, providing different electrical and optical device characteristics.

### 2. Boron-containing TADF emitters

**Figure 2:**
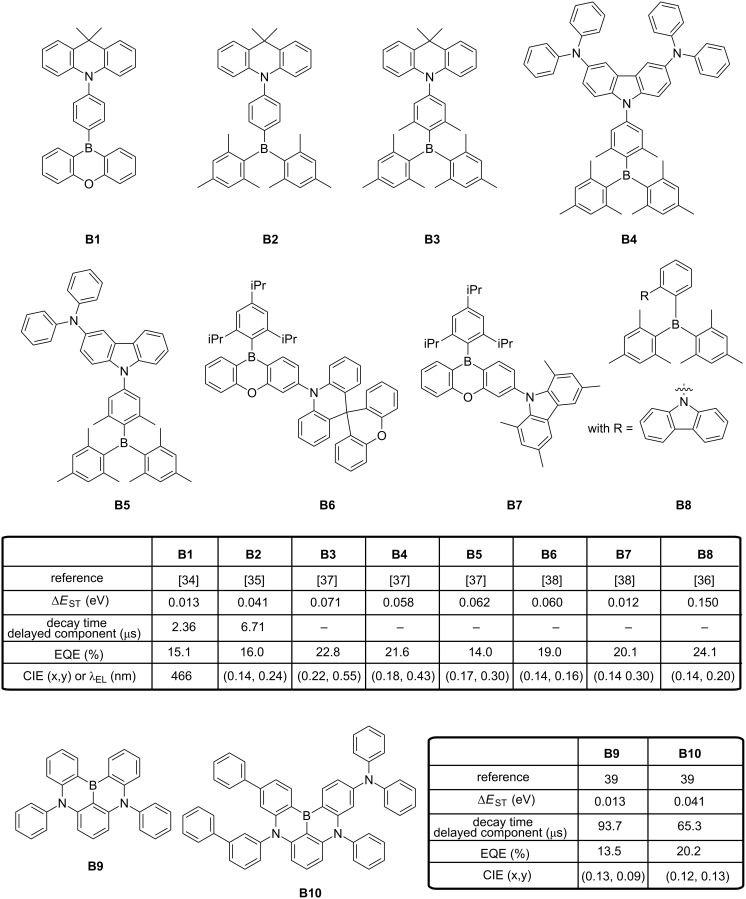
Boron-containing TADF emitters **B1**–**B10**.

Boron-containing molecules have been extensively investigated in organic electronics [31] as these materials are characterized by a remarkable electron mobility resulting from the presence of a vacant p-orbital on the boron atom [32–33]. Triarylboron compounds are also strong electron acceptors, justifying that numerous groups developed TADF emitters using triarylboron moieties as acceptors. As possible donors, diarylamines have often been proposed (carbazole, triphenylamine, carbazole/triphenylamine hybrids, 9,9-dimethyl-9,10-dihydroacridine), as exemplified in Figure 2 [34–36]. In **B1** and **B2**, isolation of the two parts was obtained by linking the 10*H*-phenoxaborin unit or the dimesitylphenylboron moiety to the 9,9-dimethyl-9,10-dihydroacridine part through a phenylene bridge substituted at the 1,4-positions. By mean of steric repulsions occurring between the hydrogen atoms of the aromatic π-bridge and those of the neighbouring electron-donating and accepting parts, an effective spatial separation of the HOMO and LUMO levels could be obtained, resulting in the rotation of the two end-groups relative to the plane of the central aromatic ring. A dihedral angle of 51.8° was found between the phenylene and the 10*H*-phenoxaborin unit in **B1**, increasing to 88.4° for the dihedral angle between the phenylene and the 9,9-dimethylacridane unit in **B2**. Δ*E*_ST_ values of 0.013 eV and 0.041 eV were experimentally determined for **B1** and **B2**, respectively, calculated from the difference existing between the onset of the fluorescence and the phosphorescence emission. The decay time of the delayed component of luminescence was determined as being 2.36 μs and 6.71 μs for **B1** and **B2**, respectively. When evaluated in multilayered OLEDs, a blue electroluminescence (EL) peaking at 466 nm and 479 nm, an external quantum efficiency (EQE) of 15.1% and 16.0% were obtained for **B1** and **B2**, respectively, indicating the substantial contribution of the triplet excitons to the luminescence.

Interestingly, compared to **B2**, the introduction of two additional methyl groups in the phenyl part **(B3**) resulted in a clear bathochromic shift of the EL, OLEDs emitting a green light peaking at 502 nm [37]. A blue shift of the emission and sky-blue OLEDs could only be obtained with this acceptor by replacing the electron-donating 9,9-dimethyl-9,10-dihydroacridinyl unit of **B3** by a bis(diphenylamino)carbazole group in **B4** or a diphenylaminocarbazole unit in **B5**. The outstanding EQE of 21.6% could be attained for the sky-blue **B4**-based devices. Still based on the combination of acridan and 10*H*-phenoxaborin units, a complete isolation of the two units could be realized in **B6** by directly functionalizing the 10*H*-phenoxaborin core with a spiro-type acridan group [38]. Using this strategy, pure blue OLEDs exhibiting an EQE of 19.0% and Commission Internationale de l’Eclairage (CIE) coordinates of (0.14, 0.16) were obtained with **B6**. Comparable performances were determined for **B7** (20.1%, (0.14, 0.16)), comprising the sterically demanding tetramethylcarbazole. In these two structures, a large dihedral angle arising from steric repulsions between hydrogen atoms in the *peri*-position of **B6** and from the presence of methyl groups at the 3,6-positions of 1,3,6,8-tetramethylcarbazole in **B7** could be obtained. In fact, the substitution at the 3,6-position of carbazole could maintain a large dihedral angle in **B7** whereas the two methyl groups at the 1,8-positions were introduced for a higher electrochemical stability of the carbazole donor. Finally, by modifying the connectivity between the donor and acceptor in **B8**, a record-high EQE of 24.1% could be realized for pure-blue OLEDs (0.139, 0.150) close to the National Television Standards Committee standard (NTSC) blue values of (0.14, 0.08) [36]. Upon *ortho*-substitution of the dimesitylphenylboron acceptor with a carbazole, a mutual steric hindrance could be exerted between the donor and the acceptor resulting in the large dihedral angle of 72.6°. A S_1_–T_1_ energy splitting of 0.13 eV could be also experimentally determined for **B8**. Interestingly, the outstanding EL characteristics of **B8**-based devices were assigned to the large contribution of the delayed fluorescence (61%) in the overall luminescence decay of **B8**. A pure blue emission could also be realized by totally blocking the structure, what was done with **B9** and **B10** in which two of the three aromatic rings of triphenylamine were connected to the boron center [39]. By elongating the π-conjugation of the electron-donating group in **B10** compared to **B9**, a more delocalized HOMO level could be generated, resulting in a greater intramolecular charge transfer and an increase of the oscillator strength. As a result, EQE of corresponding OLEDs increased from 13.5% (459 nm, (0.13, 0.09)) for **B9**-based devices to 20.2% (467 nm, (0.12, 0.13)) for **B10**-based devices. If the electron-to-photon conversions are remarkable, none of the OLEDs could reach the brightness of 1000 cd/m^2^ owing to a dramatic efficiency roll-off. Precisely, the efficiency roll-off determined for **B9**- and **B10**-based devices was determined as originating from an imbalanced charge transportation and the presence of bimolecular quenching processes occurring at high current density such as triplet–triplet annihilation and exciton–polaron annihilation.

### 3. Diphenylsulfone-based emitters

**Figure 3:**
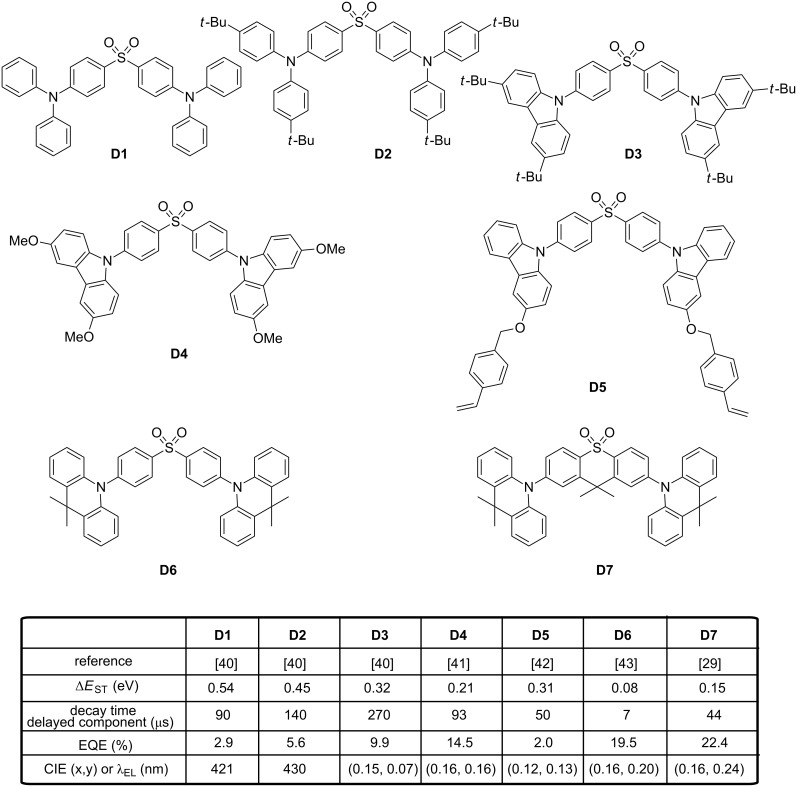
Diphenylsulfone-based TADF emitters **D1**–**D7**.

Concerning the design of blue TADF emitters, diphenylsulfone is the third most widely studied acceptor in the literature, followed by triarylboron and triazine derivatives. In this field, the contribution of the Adachi’s group is remarkable. The first report mentioning a pure blue emission with a diphenylsulfone derivative was reported in 2012 [40]. By a careful control of the π-conjugation length between the donor and the acceptor, **D3**-based OLEDs producing a deep blue emission with CIE coordinates of (0.15, 0.07) were fabricated (see Figure 3). Examination of the phosphorescence spectra of **D1**–**D3** at 77 K revealed their T_1_ states to be ^3^ππ* states centred on their electron-donating parts. Δ*E*_ST_ values of 0.54, 0.45 and 0.32 eV were, respectively, determined for **D1**–**D3**. Changes in Δ*E*_ST_ were explained as follow: By introducing *tert*-butyl groups on the diphenylamine unit, the electron donating ability in **D2** was reinforced compared to **D1**, red-shifting the charge transfer (CT) band and lowering the CT energy as well as Δ*E*_ST_. By replacing the diphenylamine unit of **D1** by a *tert*-butyl-substituted carbazole unit in **D3**, the ^3^ππ* state was considerably destabilized, raising its energy level and decreasing Δ*E*_ST_. Parallel to this, a greater separation of the HOMO and LUMO orbitals was evidenced by theoretical calculations for **D3**, as a result of a larger dihedral angle (49° instead of 32° for **D1** and **D2**), resulting in a smaller energy difference between the singlet and triplet excited states. As expected, the contribution of the slow decay component in the luminescence of **D1**–**D3** decreased with increasing ΔE_ST_, almost disappearing for **D1**. While using **D1**–**D3** as dopants for multilayer OLEDs, maximum EQEs of OLEDs coincide the order previously determined for the proportion of the delayed component in the total emission of **D1**–**D3** with the EQE (**D1**) < EQE (**D2**) < EQE (**D3**) (2.9%, 5.6% and 9.9% for **D1**–**D3**, respectively). If **D3** displayed the best EQE for the series, a dramatic efficiency roll-off at high current density was observed, as the result of a long TADF lifetime (270 μs). This issue was addressed with **D4** [41]. By replacing the *tert*-butyl groups of **D3** by methoxy groups in **D4**, a significant decrease of Δ*E*_ST_ was obtained (0.21 eV instead of 0.32 eV for **D3**), reducing the TADF lifetime and efficiency roll-off. More precisely, the higher electron-donating ability and the longer conjugation length of the 3,6-dimethoxycarbazole compared to the 3,6-di-*tert*-butylcarbazole lowered the S_1_ state and to a greater extend the T_1_ state of **D4**, furnishing in turn a molecule with a smaller Δ*E*_ST_ than **D3**. Jointly, due to the reduction of Δ*E*_ST_, a TADF lifetime of 93 μs was determined for **D4**, far from the value measured for **D3** (270 μs). When tested in a similar device structure than that previously used for **D3**, a maximum EQE of 14.5% and a smaller efficiency roll-off was evidenced for **D4**-based devices, attributed to the smaller Δ*E*_ST_ and the shorter TADF lifetime. Recently, a thermally cross-linkable and solution-processable version of **D4**, i.e., **D5** was reported in the literature [42]. If the strategy is appealing, the final EL performances of **D5**-based OLEDs were far from that obtained with vacuum-processed OLEDs and a maximum EQE of only 2.0% could be reached. Following the basic rule of molecular design consisting in maximizing the dihedral angle to minimize Δ*E*_ST_, substitution of diphenylsulfone by 9,9-dimethyl-9,10-dihydroacridine resulting in an almost orthogonality of the two groups in **D6** as a dihedral angle as large as 89° could be determined between 9,9-dimethyl-9,10-dihydroacridine and the connected phenyl ring of the diphenylsulfone unit [43].

A significant reduction of the TADF lifetime (≈7 μs) and a small Δ*E*_ST_ of 0.08 eV were measured for **D6**, favorable to the fabrication of highly emissive blue OLEDs. Devices fabricated with **D6** furnished a maximum EQE of 19.5% and maintained the high EQE of 16% at 1000 cd/m^2^ with a satisfactory color purity of coordinates (0.16, 0.20). Recently, high-performance TADF based hybrid WOLEDs employing **D6** as the blue emitter were successfully fabricated [44]. Interestingly, WOLEDs showed excellent device characteristics with an EQE of 23.0%, a current and power efficiency of 51.0 cd/A and 51.7 lm/W, respectively. These performances are among the highest values reported to date for hybrid WOLEDs using a TADF material as the blue emitter. Derivative **D6** was also examined in the context of undoped OLEDs [45]. Undoped OLEDs are more attractive than their doped analogues due to an easier fabrication process, a higher reproducibility and reliability. With regards to the highly twisted structure of **D6** and the presence of methyl groups on the 9,9-dimethyl-9,10-dihydroacridine units, this molecule proved to be also nearly insensitive to the concentration, showing an emission maximum for the neat film at 470 nm which is almost similar to that obtained for a 10 wt %-doped mCP film (462 nm where mCP stands for *m*-*bis*(*N*-carbazolyl)benzene). Parallel to this, the fluorescence and TADF lifetime were almost the same for both the doped and undoped film, making **D6** a candidate applicable for the design of undoped OLEDs. Trilayered undoped OLEDs fabricated with **D6** displayed a sky-blue emission peaking at 480 nm, with an EQE of 19.5% at a luminance of 100 cd/m^2^, slightly red-shifted compared to the emission observed for doped OLEDs. Clearly, the specific design of **D6** and its highly twisted structure efficiently weakened the π–π-stacking interactions, providing a general design rule for the elaboration of TADF emitters insensitive to the concentration. Belonging to the same family of structure than **D6**, **D7** that derives from the 9,9-dimethylthioxanthene-*S,S*-dioxide structure provided a better color purity (465 nm, (0.16, 0.24) for **D7** instead of 480 nm for **D6**) and a higher EQE (22.4% for **D7** instead of 19.5% for **D6**) than **D6** by optimizing the architecture of the doped EML [29]. By selecting the host of appropriate polarity, the combination of **D7** with the correct host could minimize the RISC barrier, optimize the RICS rate and thus maximize the TADF efficiency. While combining the blue TADF emitter **D7** with a green and an orange TADF emitter, all-TADF white OLEDs with 16% EQE could be fabricated [30].

### 4. Triazine–pyrimidine based emitters

**Figure 4:**
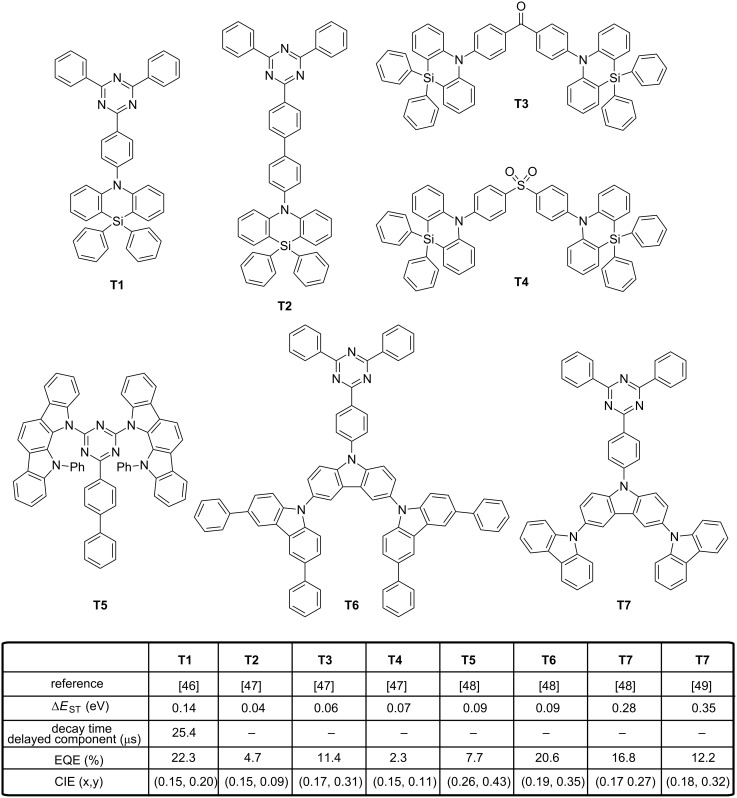
Triazine-based TADF emitters **T1**–**T3, T5**–**T7 and** azasiline derivatives **T3** and **T4**.

Among possible electron acceptors, another structure has been extensively regarded as an adequate electron acceptor for the design of blue TADF emitters and this structure is the triazine unit. When combined with the azasiline donor, OLEDs displaying the unprecedented EQE of 22.3% were obtained [46]. As specificity, azasiline is a 6-membered heterocycle comprising a silicon atom introduced instead of a carbon atom to enlarge the HOMO–LUMO gap and lower the HOMO level. Due to the sp^3^ hybridization of the silicon atom, two phenyl rings can be introduced on the silicon-bridged structure providing bulkiness and rigidity to the donor. Intermolecular interactions are thus efficiently prevented and the conformation disorder drastically reduced. When used as electron donor in **T1**, a Δ*E*_ST_ of 0.14 eV was determined experimentally, with a TADF lifetime of 25.4 μs and a 13:87 ratio between the prompt and delayed fluorescence. OLEDs fabricated with **T1** and a mCP:TSPO1 cohost (with TSPO1 = diphenyl-4-(triphenylsilyl)phenylphosphine oxide) furnished a blue emission peaking at 463 nm, with CIE coordinates of (0.149, 0.197) and a low efficiency roll-off. Another key and general design rule for obtaining a small Δ*E*_ST_ consists in the physical separation of the donor and the acceptor by elongating the spacer that couples the two partners. Following this recommendation, an additional phenyl ring was introduced between the donor and the acceptor in **T2**, providing the extended version of **T1** (see Figure 4) [47]. As expected, the phenyl ring increased the separation of the HOMO and LUMO orbitals, such that Δ*E*_ST_ decreased. A value as low as 0.04 eV was experimentally determined for **T2**. In doped devices, **T2** demonstrated an EL efficiency of 4.7% with a deep blue emission (0.151, 0.087) approaching the NTSC blue standard (0.14, 0.08). However, a comparison with the previous EL performance evidenced that EQEs obtained with **T2** are 5 times lower than that obtained with **T1**, despites the more favorable S_1_–T_1_ energy splitting. This problem is commonly observed if the isolation of the electron-donating and electron-accepting parts is obtained upon extension of the distance between the two moieties. Indeed, as a consequence of this strategy, a weaker intramolecular charge transfer takes place and a reduction of the oscillator strength in the D–A diad is observed, resulting in a drastic reduction of the PLQY and thus of the external quantum efficiency. In the same study, authors examined the case of two TADF emitters based on a donor–acceptor–donor (D–A–D) structure, i.e., **T3** and **T4**, where azasiline was used as the donor and diphenylsulfone or benzophenone as the acceptors. Here again, the higher twisted molecular structure of **T4** was beneficial in terms of Δ*E*_ST_, color purity and EL performances. Thus, the higher internal torsion of **T4** furnished OLEDs with a deeper blue emission (0.154, 0.107) than devices fabricated with **T3** (0.174, 0.310). Even if the EQE of **T4**-based devices was lower than that of **T3**-based devices (2.3% for **T4**-based OLEDs instead of 11.4% for **T3**-based devices), it is attributable to the higher color purity of **T4**-based devices and not to differences of Δ*E*_ST_ (0.07 eV and 0.06 eV for **T3** and **T4**, respectively). Azasiline is a promising electron donor but examples of blue TADF emitters are still scarce. The opposite situation is found for carbazole, which has long been considered as an excellent donor and a large variety of blue TADF emitters have been designed on the basis of this scaffold. At least 19 examples of blue TADF emitters can be cited, the molecules differing by the strategy used to connect the donor(s) to triazine. However, contrarily to azasiline that possesses a six-membered central ring, carbazole only possesses a five-membered central ring, inducing a deviation of the two adjacent aromatic rings. As a result, carbazole is not capable to induce the same encumbrance as that of azasiline by inducing smaller steric effects and the substitution of the 1,8-positions is often required to maintain a large dihedral angle.

**Figure 5:**
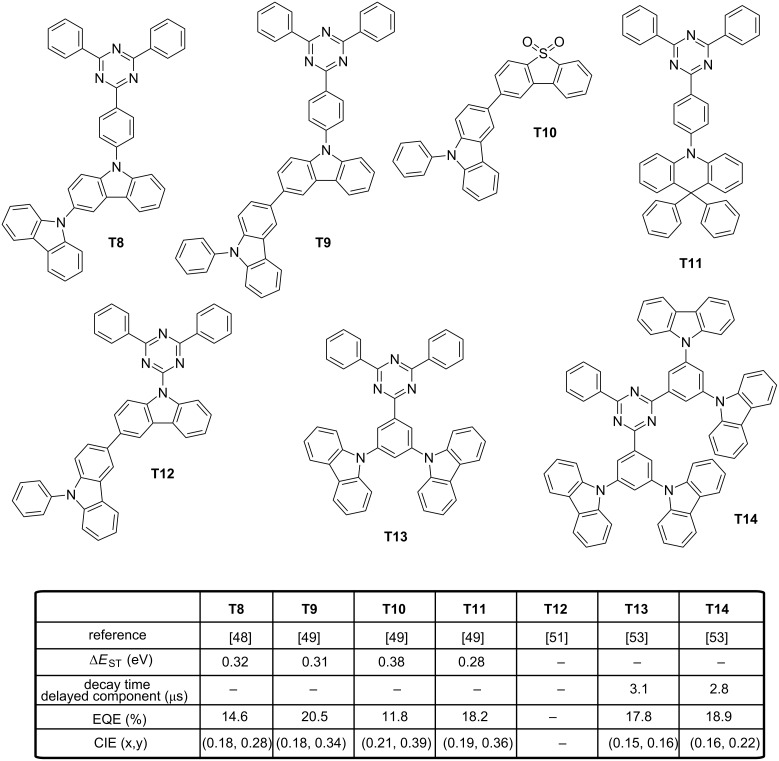
Triazine-based TADF emitters **T8**, **T9**, **T11**–**T14** and carbazole derivative **T10**.

As interesting design rules, Adachi determined that the extension of the electronic delocalization of both the HOMO and LUMO energy levels could greatly increase the rate of the radiative decay by inducing a large oscillator strength while lowering Δ*E*_ST_, even for emitters for which only a small overlap between the two wavefunctions is observed [48]. These findings constitute a second guideline for the molecular design of TADF emitters that can address the distance and the reduction of the oscillator strength issue previously mentioned. To establish this, a series of molecules **T5**–**T8** with varying length of the π-conjugated system for the donating part was investigated. Thus, for **T5** and **T6**, a similar Δ*E*_ST_ value of 0.09–0.12 eV was experimentally determined for the two emitters. However, significant differences were determined for their PLQYs and values of 0.1 and 0.7 were measured for **T5** and **T6**, respectively. By theoretical calculations, the oscillator strength of **T6** was found to be 13.6 times greater than that of **T5**, supporting the enhanced luminescence of **T6** by the higher delocalization of its HOMO level. This trend was confirmed by keeping the acceptor constant in **T6**–**T8**. An increase of Δ*E*_ST_ while reducing the possible electronic delocalization over the electron-donating part was clearly evidenced going from **T6** to **T8**. In OLEDs, EL performances followed the same trend, with the highest EQE obtained with **T6** (EQE = 20.6%) and the lowest one with **T8** (EQE = 14.6%). A lower color purity was obtained for **T6**-based devices (λ_EL_ = 487 nm) compared to **T7** and **T8** (λ_EL_ = 478 and 477 nm, respectively) [22]. A worse result was obtained for **T5** that produced a blue-green EL at 506 nm. Recently, an extensive work was devoted to examine the degradation mechanisms in blue TADF OLEDs and **T7** was revisited in this context [49]. The synergy of an electro-oxidation process together with a photo-oxidation was determined as playing a critical role in the degradation of blue TADF emitters. In fact, a parallel can be easily done with the treatment of wastewater, where pollutants are removed from water by combining a photochemical and an electrochemical process [50]. During this study, the localization of the triplet spin density was found determinant for the stability of blue TADF emitters. To evidence this, four emitters (**T7**, **T9**–**T11**) exhibiting the same S_1_ and T_1_ energy levels, the same TADF lifetimes but differing by the distribution of the triplet spin densities were examined (see Figure 4 and Figure 5). Notably, for **T9**, the triplet spin density was found to be mainly localized on the bicarbazole donor, whereas for **T7** and **T10**, the triplet spin density is localized on their acceptor fragment. To end, the triplet spin density of **T11** is delocalized over the entire structure. While examining the device lifetime, **T9**-based devices had the longest device lifetime (32 hours), far from **T10**-, **T7**- and **T11**-based OLEDs (1.4 h, 2.8 h and 0.9 h, respectively), demonstrating the higher stability of the emitters with a triplet spin density centered onto the donor unit. In another study, an analogue of **T9**, i.e., **T12**, differing by the removal of a phenyl ring between the carbazole and the triazine units proved once again the crucial role of the oscillator strength in the photophysical properties [51]. Notably, major differences in the separation of their HOMO and LUMO energy levels were determined by theoretical calculations. An overlap of the two electronic wavefunctions was detected for **T9** whereas the two orbitals are strongly localized in the case of **T12**. Resulting from this marked localization in **T12**, a smaller variation of the electronic density upon excitation is expected, reducing the oscillator strength and the PLQY. When tested in devices, only a green-blue emission was obtained with **T12** (see Figure 5) [52]. The Influence of the oscillator strength on OLEDs characteristics could also be evidenced while comparing **T13** and **T14** [53]. Molecular orbital calculations performed on **T13** and **T14** showed the two molecules to exhibit a similar electronic distribution, what was confirmed by UV–visible and photoluminescence (PL) spectroscopy. Only a slight red shift of the absorption was detected for **T14** as the result of the strengthened donating ability of the dicarbazolylphenyl moieties. Similarly, almost identical Δ*E*_ST_ were determined with values of 0.25 and 0.27 eV for **T13** and **T14**, respectively). As it could be anticipated, **T14** furnished slightly better EL performances (18.9%) compared to that measured for **T13** (17.8%), due to its more extended donating part but also owing to its higher PLQY. Conversely, the color purity was higher for **T13**-based devices (λ_EL_ = 459 nm) instead of 467 nm for **T14**-based devices. However, a remarkable device stability was demonstrated for **T14**-based OLEDs, 80% of the initial luminance being retained after 52 hours. This value was reduced to only 5 hours for **T13**-based OLEDs. A comparison established with an iridium complex, i.e., tris[1-(2,4-diisopropyldibenzo[*b*,*d*]furan-3-yl)-2-phenyl-1*H*-imidazole]iridium(III) (Ir(dbi)_3_) evidenced the relevance of the TADF approach, as a device lifetime of only 18 hours was found while operating OLEDs in the same conditions. The spatial separation of the electron-donating part from the electron-accepting moiety by elongating the spacer has already been discussed and the drawbacks evoked.

Minimization of the electron density overlap can also be realized by means of an *ortho*-phenyl linkage, enabling to maintain the donor in proximity of the acceptor.

**Figure 6:**
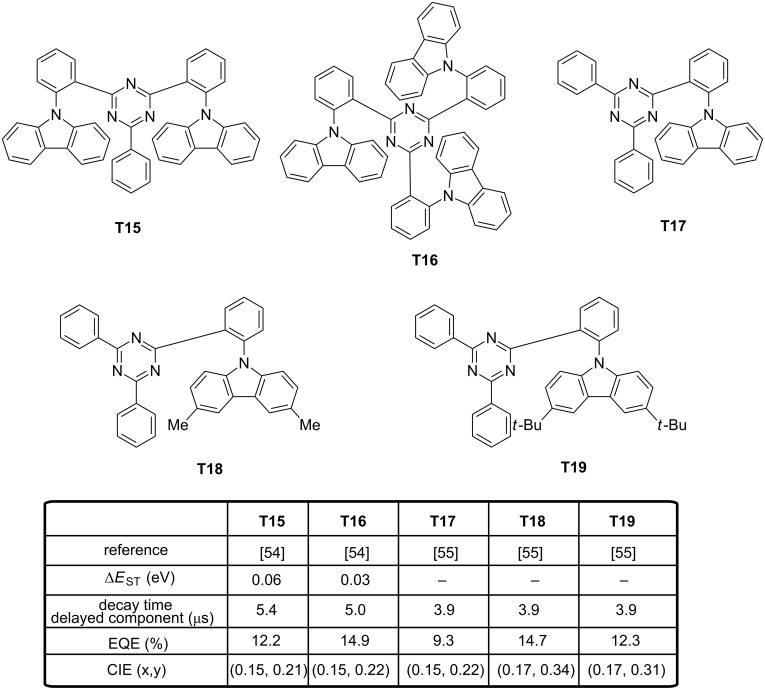
Triazine-based TADF emitters **T15**–**T19**.

In this situation, one aromatic ring of the donor and/or the acceptor is substituted at the 1,2-positions, generating a highly-twisted structure. Five blue TADF emitters **T15**–**T19** were designed on this basis (see Figure 6). By increasing the number of carbazoles in **T16** compared to **T15**, a decrease of Δ*E*_ST_ was logically observed (0.06 eV for **T15** and 0.03 eV for **T16**) [54]. A large torsion angle of 66° and 67° were, respectively, determined by theoretical calculations for **T15** and **T16**, favorable to the separation of the two orbitals. In devices, a remarkable enhancement of the EL performances was realized by increasing the number of carbazole units. Thus, a maximum EQE of 12.2% was realized with **T15**, whereas an EQE of 16.5% was determined for **T16**-based devices.

This enhancement can also be attributable to an increase of the oscillator strength from **T15** to **T16**, the number of donors being increased. The low efficiency roll-off of **T16**-based devices was assigned to the specific design of the emitter, with the triazine acceptor being totally surrounded by carbazoles. As a result, triplet–triplet annihilation by the Dexter mechanism could be efficiently prevented, enabling to maintain high efficiencies at high current density. Although the number of carbazole units increased, no modification of the EL position was detected, the emission peaking at 467 and 468 nm for **T15**- and **T16**-based devices. In the same spirit, other authors examined the possible impact of the substitution pattern of the carbazole unit on the photophysical properties.

**Figure 7:**
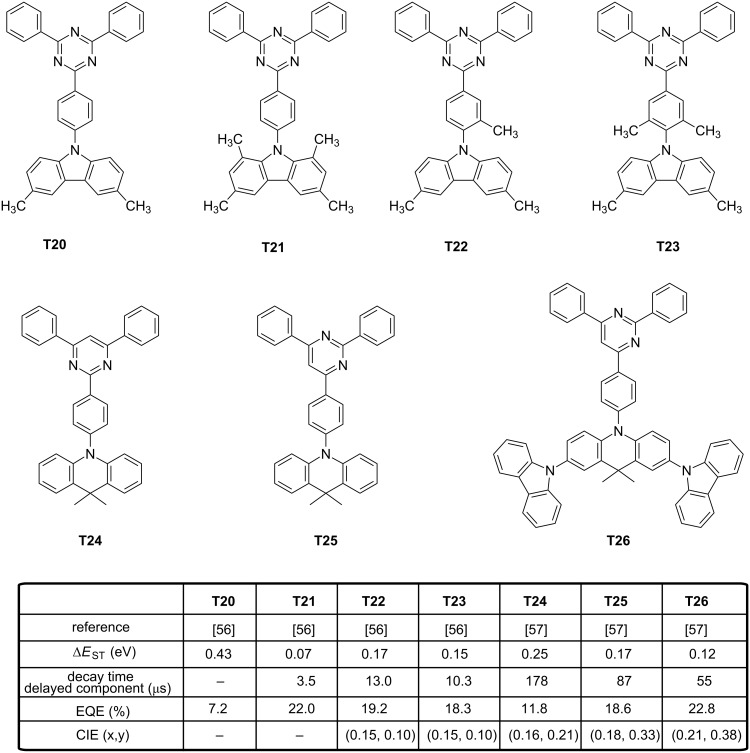
Triazine- and pyrimidine-based TADF emitters **T20**–**T26**.

While maintaining the same number of carbazole units on the emitter and by varying the substitution pattern of the carbazole core, only a weak influence on the EL characteristics was evidenced [55]. In fact, performances only varied by their differences of PLQYs (16.7%, 50.5% and 43.0% for **T17**, **T18** and **T19**, respectively), the three molecules exhibiting similar photophysical properties (Δ*E*_ST_, emission wavelength and decay times of the delayed emission). Recently, a potential alternative to the *ortho*-substitution of the triazine acceptor by carbazole moieties was examined, consisting in introducing methyl groups in the proper position of the triazine or the carbazole moieties [56]. By changing the methyl group positions, optical properties of **T20–T23** were not significantly modified, contrarily to their Δ*E*_ST_ (see Figure 7). In fact, the authors evidenced the introduction of methyl groups at the 1,8-positions of carbazole to be harmful for producing a deep-blue emission whereas the substitution of the central phenyl ring by methyl groups could provide the same molecular twist than the 1,8-substitution of carbazole while maintaining a large optical bandgap. In fact, dihedral angles of 49.9, 86.8, 71.4 and 82.4° were determined by density functional theory (DFT) calculations between the donor plane and the acceptor plane for **T20**–**T23**, respectively. Due to its lesser twisted structure and based on the design rule previously evoked (orthogonality between the donor and the acceptor is researched to isolate the two groups), **T20** showed the higher Δ*E*_ST_ of the series. Theoretical calculations clearly evidenced for **T20** the HOMO level to extend to the neighbouring phenylene bridge, adversely affecting Δ*E*_ST_. Conversely, the large dihedral angle of **T21**–**T23** contributed to spatially separate the HOMO from the LUMO orbitals. By electrochemistry, an appreciable reduction of the oxidation potential was detected (+0.87 V) for **T21** which is substituted at the 1,8-positions of the donor whereas **T20**, **T22** and **T23** exhibited the same oxidation potentials (+0.97 V). By PL, T_1_ states of **T20**, **T22** and **T23** proved to be ^3^LE states whereas a ^3^CT state was found for **T21**.

**Figure 8:**
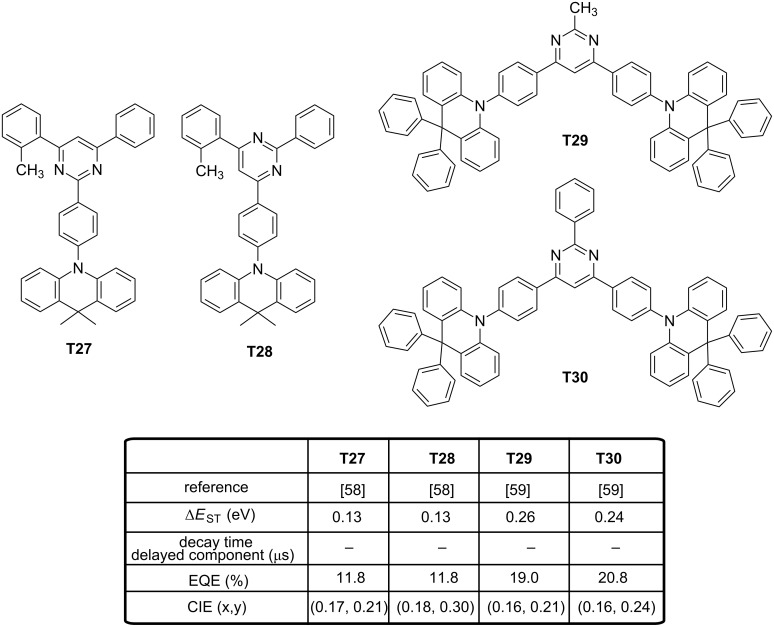
Pyrimidine-based TADF emitters **T27**–**T30**.

To evidence this, examination of the phosphorescence spectra of **T20–T23** in a frozen toluene matrix at 77 K revealed for **T20**, **T22** and **T23** to exhibit well-resolved vibrational structures, demonstrating their T_1_ states to be ^3^LE states. Conversely, only a broad spectrum was obtained for **T21**, and its triplet state was assigned to a ^3^CT state. Precisely, by its large dihedral angle, **T21** differs from **T20**, **T22** and **T23** by the order of its orbitals, ^3^LE and ^3^CT being inverted in this case. Analysis of the transient PL decay curves showed **T20** to exhibit a negligible delayed fluorescence as a result of large Δ*E*_ST_. On the opposite, prompt and delayed fluorescence components were clearly evidenced for **T21**–**T23**. Lifetimes of the delayed components for **T21**–**T23** were 3.5, 13.0 and 10.3 μs, respectively. Due to the inability of **T20** to upconvert its electrons from the triplet to the singlet state, **T20**-based device could only reach an EQE of 7.2%. On the opposite, maximum EQEs of 22.0, 19.2 and 18.3% were obtained for **T21**–**T23**-based devices, with CIE coordinates of (0.148, 0.098) and (0.150, 0.097) for **T22**- and **T23**-based devices, respectively. As anticipated, a lower color purity was obtained for **T21**-based devices resulting from its lower oxidation potential. Recently, a significant enhancement of blue OLED performances was obtained by replacing the triazine acceptor by a 2,4,6-triphenylpyrimidine unit in donor-acceptor-based TADF emitters [57]. Considering that the electron acceptor is not symmetrical anymore, positions of the nitrogen atoms will significantly influence the distribution of the electronic cloud and potentially the overlap with the HOMO level. Examination of the electronic properties of **T24** revealed the HOMO and the LUMO levels are located on both the donor and acceptor part, respectively, without any contribution of the phenyl linker. Another situation was found for **T25** and **T26** since the LUMO predominantly extends on both the acceptor and the phenyl ring which is between the donor and the 4,6-diphenylpyrimidine fragment. Due to the smaller overlap of the two wavefunctions, a weaker intramolecular charge transfer was attended, enabling to provide an emission in the blue or sky-blue region. Optical properties were evaluated in solution confirming this trend, with an emission at 455, 476, and 496 nm for **T24**–**T26**, respectively. Major differences could be found in the contribution of the delayed component in the luminescence decay. Following the trend determined for the intramolecular charge transfer, a regular increase of the prompt component in the overall decay of the three emitters was found, evidencing the up-conversion of the triplet excitons to the singlet ones. The best EQE was obtained for **T26**-based devices (22.8%) consistent with the higher delocalization of its electron-donating part, its smaller Δ*E*_ST_ and the higher contribution of the delayed component in the overall luminescence decay. A regular decrease of the EQE was observed for **T25**-based devices (18.6%) and **T24**-based devices (11.8%), confirming the absence of delayed fluorescence for the last emitter and the reduction of the strength of ICT interactions. Interestingly, the EQE reported for **T26**-based devices is among the best so far reported for blue OLEDs. Attesting the interest of the community for this new acceptor, other authors developed quasi-simultaneously a structure–performance relationship with **T24**, **T25** and **T27**–**T28** (see Figure 8) [58]. The choice of pyrimidine as the electron acceptor was notably justified by authors due to the easier synthesis of the central core and a versatile peripheral substitution. Additionally, compared to triazine, the LUMO level of pyrimidine is slightly destabilized, facilitating the access to wide bandgap materials. In this work, a more intriguing behaviour was found even for **T24** and **T25** that have just been discussed above since mechanochromic properties were evidenced for the four emitters. Based on photophysical investigations, the presence of two different packing modes in the solid state were proven. When tested in OLEDs, no clear conclusions could be deduced as results of opposite trends were detected. Thus, if the EQE of **T24**-based OLEDs was lower than that determined for **T27**-based OLEDs (7.2% and 11.8%, respectively), the opposite trend was found with **T25** and **T28** (12.6% and 11.8%, respectively). Only the influence of the symmetrical or the unsymmetrical substitution of the pyrimidine acceptor by the donor was evidenced, following the conclusions of previous authors.

**Figure 9:**
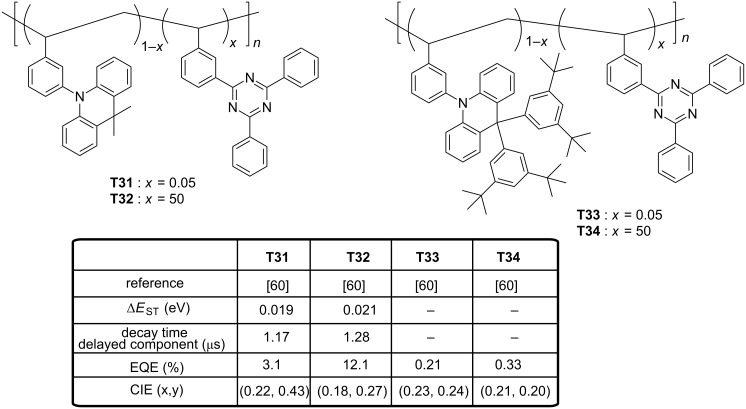
Triazine-based TADF polymers **T31**–**T32**.

Finally, two D–A–D triads comprising the 9,9-diphenyl-9,10-dihydroacridine donor were reported in 2016 [59]. Here again, existence of relatively large dihedral angles of 82–87° between the donor unit and the nearby phenylene linker for **T29** and **T30** was confirmed by quantum chemical calculations. Resulting from the almost perfect orthogonality, a good confinement of the electronic density of the two orbitals was obtained with a HOMO level predominantly located on the donor and a distribution of the LUMO over the central pyrimidine acceptor core and the adjacent phenylene linkers small Δ*E*_ST_ were determined (0.16 and 0.15 eV for **T29** and **T30**, respectively), indicative of reduced electronic correlations between frontier orbitals and accounting for their high performance. Indeed, EQEs of 19.0 and 20.8%, an EL at 468 and 472 nm were, respectively, determined for **T29** and **T30**. However, the efficiency roll-off was quite severe and this drawback was assigned to the relatively long exciton lifetimes of **T29** and **T30** in doped films (330 and 210 μs, respectively). Recently, an original strategy to combine the electron-donating 9,9-dimethyl-10-phenylacridan with the electron-accepting 2,4,6-triphenyl-1,3,5-triazine was reported under the form of random copolymers derived from a polystyrene (**T31**–**T34**, see Figure 9) [60]. Contrarily to the classical TADF materials in which the electron donor is connected to the acceptor, interactions between the two moieties occur by mean of a through-space charge transfer (TSCT). Polystyrenes of different compositions **T31**–**T34** were examined, varying by the acceptor content (5 or 50 wt % of acceptor) and the donor units, i.e., 9,9-dimethyl-10-phenylacridan or 9,9-bis(3,5-di-*tert*-butylphenyl)-10-phenylacridan. Precisely, effect of the steric hindrance on TADF properties of the polymers was investigated by introducing a steric hindrance on the electron donor. Use of polystyrene to generate EL materials is counterintuitive due to its inherent insulating character, but EL polymers substituted with iridium complexes have previously been studied in the literature, evidencing the pertinence of the strategy [61].

In this case, charge transport properties are provided by the substituents attached to the polymer chain. As main finding of this work, the detrimental effect of the steric hindrance was demonstrated, no TSCT effects and no TADF features were detected for **T33** and **T34**. Conversely, for the less hindered polymers, a delayed fluorescence could be evidenced for the two polymers **T31** and **T32**, with a ratio for the prompt/delayed component of 13/87, respectively. Δ*E*_ST_ values of 0.019 (**T31**) and 0.021 eV (**T32**) were also determined by examining the fluorescence and phosphorescence spectra. Interestingly, the bluest EL emission (472 nm) was obtained for the polymer only containing 5 wt % of acceptor **T31**, with an EQE peaking at 12.1% for these solution-processed OLEDs, what is remarkable. Conversely, a less blue emission was obtained for **T32**, the emission peaking in the blue-green region (497 nm).

### 5. Phenoxaphosphine oxide and phenoxathiin dioxide derivatives

**Figure 10:**
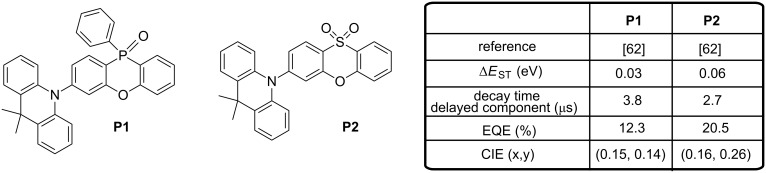
Phenoxaphosphine oxide and phenoxathiin dioxide-based TADF emitters **P1** and **P2**.

Recently, phenoxaphosphine oxide and phenoxathiin dioxide have gained interest as electron acceptors since the first report mentioning their use as acceptors was published by Lee et al. in 2016 [62]. Prior to this work, phenoxaphosphine oxide derivatives were mostly studied for the design of flame-retardants [63] or as chiral molecules for fullerene recognition [64–66]. Similarly, the scope of applications of phenoxathiin dioxide ranged from antimicrobial activity [67] to the use as inhibitor for *Hepatitis C* virus infection [68]. Here, in the context of OLEDs, Lee et al. reported two blue TADF emitters, **P1** and **P2** (see Figure 10), containing a phenoxaphosphine oxide or a phenoxathiin dioxide acceptor covalently linked to a dimethylacridan donor.

Theoretical calculations predicted the two molecules to adopt in their optimized molecular geometries a highly twisted conformation, what is a requirement for a spatial separation of the HOMO and LUMO energy levels. As attended, the LUMOs of **P1** and **P2** are localized on the acceptor moieties whereas their HOMOs are mostly distributed on the donor. Separation of the frontier orbitals lead to Δ*E*_ST_ values of 0.02 (**P1**) and 0.10 eV (**P2**), which are in perfect accordance with the experimental data: Δ*E*_ST_ = 0.03 and 0.06 eV for **P1** and **P2**, respectively. Interestingly, theoretical calculations also showed the higher electron-accepting ability of the phenoxathiin dioxide moiety compared to that of the phenoxaphosphine oxide group owing to the stronger electron-withdrawing properties of the sulfone group, with a theoretical LUMO level at −1.52 and −1.24 eV for **P2** and **P1,** respectively. In multilayered devices, remarkable CIE coordinates could be realized with **P1**- and **P2**-based OLEDs ((0.15, 0.14) with **P1** and (0.16, 0.26) with **P2**), combined with high EQEs (12.3% and 20.5%, respectively). Additionally, for **P2**-based devices, the efficiency roll-off could be remarkably suppressed and an EQE as high as 13% could be maintained at the luminance of 1000 cd·m^−2^.

### 6. CN-Substituted pyridine and pyrimidine derivatives

**Figure 11:**
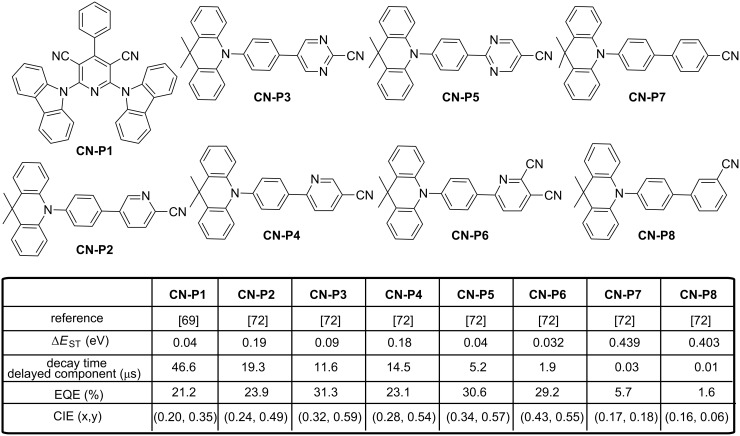
CN-Substituted pyridine and pyrimidine derivatives **CN-P1**–**CN-P8**.

In 2015, Liu et al. constructed a novel blue TADF emitter **CN-P1** comprising a carbazole donating moiety connected to a pyridine-3,5-dicarbonitrile accepting group (see Figure 11) [69]. The choice of pyridine-3,5-dicarbonitrile as acceptor was notably motivated by the outstanding charge-transport ability and the remarkable electrochemical stability of this group [70–71]. Thus, **CN-P1** had a small singlet−triplet splitting (Δ*E*_ST_ = 0.04 eV), fairish PLQY in doped films (49.7%), and a delayed decay lifetime of 46.6 μs, which suggests that it could be a promising candidate as emitter. EL performance of **CN-P1** was investigated in OLEDs with different **CN-P1** doping concentrations in *m*CP as the emitting layers. The highest EQE (21.2%) of devices was obtained at 13 wt % doping conditions. It was found that the maximum EQEs are enlarged along with the increase of doping concentration, which can be mainly attributed to the more efficient exciton utilization with a higher emitter concentration. However, EQEs decreased with the further concentration increase of **CN-P1** due to the strong interaction and aggregation between **CN-P1** molecules at high doping concentration in the emitting layer. Authors obtained EL spectra red-shifting from sky-blue (λ_max_ = 475 nm, CIE = (0.18, 0.26)) to greenish-blue (λ_max_ = 510 nm, CIE = (0.24, 0.40)) emissions by varying the doping concentration from 5 to 50 wt %. Such red shift is clearly caused by the interaction between **CN-P1** molecules at high dopant concentrations. Parallel to this, **CN-P1** molecules can also increase the polarity of the EML, thus introducing a solvatochromaticity-like shift comparable to that observed in solutions while varying the solvents polarity. The optimized device exhibited a maximum current efficiency of 47.7 cd·A^−1^, and a maximum power efficiency of 42.8 lm·W^−1^ without any light outcoupling structures, indicating that nearly 100% of excitons are harvested for light emission. Such high performance should not only be attributed to the fairish PLQY and the efficient RISC process from T_1_ to S_1_ of **CN-P1** emitter, but also owed to the reasonable high T_1_, good charge mobility, and well-matched PL spectrum of the mCP host with the **CN-P1** absorption spectrum. Still based on pyridine derivatives, Pan et al. prepared a series of twisted D–π–A type emitters based on the dimethylacridan and different CN-substituted acceptors (pyridine, pyrimidine, and benzene, see Figure 11) [72]. Theoretical calculations showed the different emitters to adopt a nearly orthogonal conformation between the donor and the central aromatic ring, interrupting the π-conjugation and localizing the HOMO level on the acridan moiety and the LUMO level on the central accepting group. The calculations also predicted a more planar phenyl–pyrimidine/phenyl–pyridine conformation (i.e., a smaller dihedral angle) in **CN-P5**/**CN-P4** and a more twisted phenyl–pyrimidine/phenyl–pyridine conformation (i.e., a larger dihedral angle) in **CN-P3**/**CN-P2**. All the DFT-optimized data were in perfect accordance with single crystal X-ray diffraction analyses. The results showed that the molecular conformations (twist angles in D-spacer-A diads) could be easily tuned by controlling the orientation of the nitrogen atom(s) in the heteroaromatic rings relative to the donor plane. In fact, two main groups of molecules were identified. Thus, **CN-P3**, **CN-P5** and **CN-P6** are characterized by a relatively small ∆*E*_ST_ of 0.032–0.090 eV, show the most pronounced contribution of the delayed component in PL with emission quantum yields for the delayed component of luminescence in the 38–44% range.

**Figure 12:**
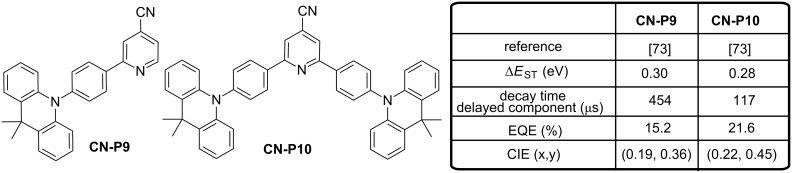
CN-Substituted pyridine derivatives **CN-P9** and **CN-P10**.

These molecules also exhibit high reverse intersystem crossing rates (*k*_RISC_ > 15 × 10^4^ s^−1^). Conversely, **CN-P2** and **CN-P4** show larger ∆*E*_ST_ (0.180 − 0.190 eV) than **CN-P3**, **CN-P5** and **CN-P6** and lower TADF contributions in PL with smaller quantum yields for the delayed component of luminescence (19–23%). Smaller RISC were also determined (*k*_RISC_ of < 8 × 10^4^ s^−1^). Finally, TADF contribution on the total luminescence of **CN-P7** and **CN-P8** was the weakest of the series (≤1%) as a result of their extremely large ∆*E*_ST_ (>400 meV). Due to the weak contribution of the TADF process, these emitters could be nearly assimilated to conventional fluorescent emitters. All light-emitting materials show lifetimes for the prompt decay component of luminescence in the 6.5–27 ns range whereas the lifetimes for the delayed decay component varied from 1.9 to 19 μs. All compounds were tested in OLED and all devices exhibited a relatively low turn-on voltage (≈2.5 V) and a low operation voltage (≈3.5–4 V for a brightness of 100 cd·m^−2^). Devices using high-PLQY emitters (PLQY = 90–100%) exhibited rather high EQEs of up to 23.1–31.3%, while **CN-P7** and **CN-P8** having the lower PLQYs gave inferior EQEs of 5.7% and 1.6%, respectively. Noticeably, emitters showing the most pronounced TADF characteristics (i.e., **CN-P6**, **CN-P3**, and **CN-P5**) furnished the remarkable EL efficiencies of 29.2% (96.3 cd·A^−1^, 105.5 lm·W^−1^), 31.3% (104.5 cd·A^−1^, 117.2 lm·W^−1^), and 30.6% (103.7 cd·A^−1^, 116.3 lm·W^−1^), respectively. On the opposite, **CN-P2** and **CN-P4** showing the less pronounced TADF characteristics exhibited similarly high PLQYs (90–92%) but lower EQEs (23–24%). Finally, **CN-P8,** in which the TADF contribution is almost inexistent, furnished the low EQE of 5.7% (this is also the material exhibiting the lowest PLQY (36%)), yet such an EQE is still significantly higher than it can be expected from a conventional non-TADF fluorescent emitter of similar PLQY (i.e., EQE can be estimated to be ≈2.5–3% at most), suggesting therefore the contribution from the delayed fluorescence in the overall EL process. Although **CN-P6**, **CN-P5**, and **CN-P3** could reach high maximum EQEs, different efficiency roll-off behaviours could be evidenced with the following order: **CN-P6** < **CN-P5** < **CN-P3**. Such a trend for the efficiency roll-off correlate well with the order of their delayed fluorescence lifetimes and their RISC decay rate values in the host film: **CN-P6** < **CN-P5** < **CN-P3** for the delayed fluorescence lifetimes and **CN-P6** > **CN-P5** > **CN-P3** for *k*_RISC_. Such correlation is also observed for **CN-P4** and **CN-P2** devices. It has been rationalized that a small delayed fluorescence lifetime (and thus effective RISC) is beneficial for faster triplet-to-singlet conversion, for reducing the triplet exciton population at higher brightness/current, and thus for reducing associated quenching mechanisms (e.g., triplet–triplet annihilation, etc.). This year, Sasabe et al*.* reported high efficiency blue OLEDs using isonicotinonitrile-based fluorescent emitters comprising 9,10-dihydro-9,9-dimethylacridine(s) as donor unit(s) [73]. The chemical structures of the two emitters **CN-P9** and **CN-P10** is given in Figure 12. While evaluating the optical and photophysical properties of the different materials, all compounds showed reasonably high PLQYs (71–79%) in the host films, with a sky-blue emission located at 489 and 495 nm for **CN-P9** and **CN-P10**, respectively. Delayed luminescence lifetimes of 453.7 µs and 116.9 µs, sufficiently small ∆*E*_ST_ of 0.30 eV and 0.28 eV to allow a RISC were also determined for **CN-P9** and **CN-P10**, respectively. Performances of the two sky-blue emitters **CN-P9** and **CN-P10** were then evaluated in OLEDs. **CN-P9**-based devices showed a sky-blue emission with CIE chromaticity coordinates of (0.19, 0.36), a low turn-on voltage of 3.1 V and an EQE of 15%. In contrast, **CN-P10**-based devices showed still a sky-blue emission with CIE coordinates of (0.22, 0.45), a low turn-on voltage of 2.9 V but an EQE peaking at 22%, resulting from its smaller ∆*E*_ST_. Considering the EQE values overcoming the 5% EQE limit for fluorescent materials, contribution of a TADF process in the overall emission of these two emitters was clearly demonstrated.

### 7. Phosphine oxide derivatives

**Figure 13:**
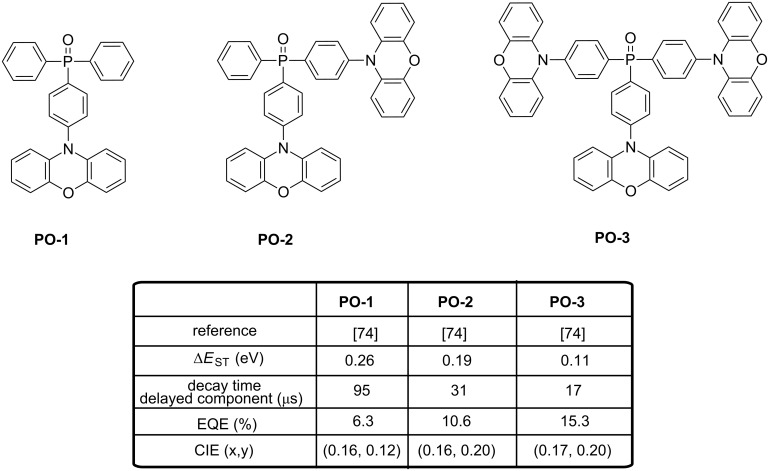
Phosphine oxide-based TADF blue emitters **PO-1**–**PO-3**.

Blue thermally activated delayed fluorescence (TADF) dyes are basically combinations of strong acceptors and weak donors. In their recent work, Duan et al. employed a weak acceptor group to construct a series of weak acceptor−strong donor (WASD)-type emitters with a phenoxazine donor [74]. The molecular structures of these fluorescent compounds, namely 4-(10*H*-phenoxazin-10-yl)phenyl)diphenylphosphine oxide (**PO-1**), bis(4-(10*H*-phenoxazin-10-yl)phenyl)phenylphosphine oxide (**PO-2**), and tris(4-(10*H*-phenoxazin-10-yl)phosphine oxide (**PO-3**) are given in Figure 13. Similar absorption spectra were measured in dilute solutions for all compounds, with three characteristic bands detected around 370, 320, and 240 nm. The first one was assigned to a n→π* transition from the phenoxazine group to the triphenylphosphine oxide group whereas the second and the third peak was attributed to π→π* transitions of the phenoxazine and the phenyl moities, respectively. A relation of proportionality was demonstrated in the intensities of the band, directly related to the number of phenoxazine groups per molecule. Almost identical PL spectra were determined for these molecules, proving the insulating character of the phosphine oxide group and the pertinence of the WASD strategy to preserve the emission color. Consistent with TD-DFT results, Δ*E*_ST_ decreased from 0.26 to 0.19 and finally 0.11 eV for **PO-1**, **PO-2** and **PO-3**, respectively. Relatively high PLQYs were also determined (45%, 57%, and 65%, for **PO-1**, **PO-2** and **PO-3**, respectively). PLQY of **PO-3**-based films were determined as 67%, higher than the values determined for **PO-2**- and **PO**-**1**-doped films. The prompt fluorescence lifetimes of **PO-1**, **PO-2**, and **PO-3** are gradually increasing from 8 to 13 to 20 ns. In contrast, the respective order of the delayed fluorescent lifetimes is reversed, at 95, 31, and 17 μs, accompanied by a gradual increase of the quantum yields of 36%, 45%, and 51%, respectively. **PO**-**1**-based OLED achieved EL emissions with peaks at 448 nm and CIE coordinates of (0.16, 0.12), corresponding to a deep-blue light. **PO**-**2**-based devices displayed a blue emission peaking at 460 nm and CIE coordinates of (0.16, 0.20).

**Figure 14:**
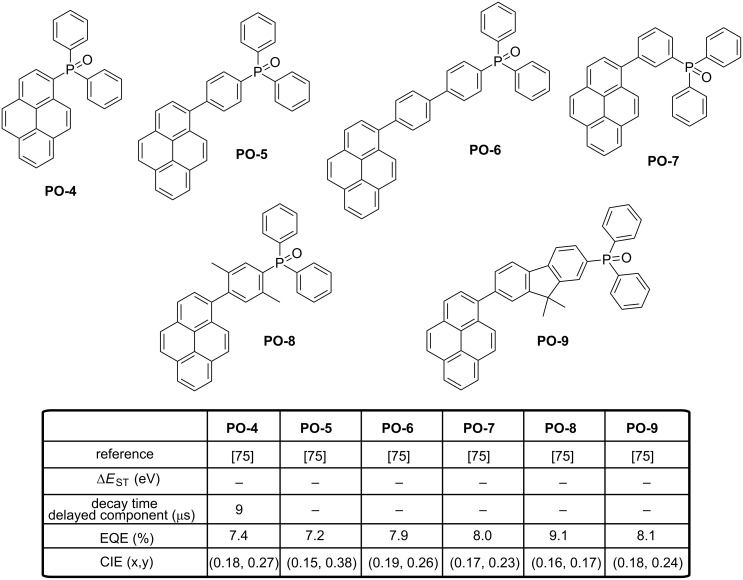
Phosphine oxide-based TADF blue emitters **PO-4**–**PO-9**.

OLEDs fabricated with **PO-3** produced a pure-blue EL emission peaking at 464 nm, an EQE up to 15.3%, a low efficiency roll-off and CIE coordinates of (0.17, 0.20). With aim at simplifying the device fabrication, other authors tried to develop emitters **PO-4**–**PO-9** specifically designed for the fabrication of non-doped OLEDs (see Figure 14) [75]. To reach this goal, the electron-transport diphenylphosphine oxide group was attached to pyrene moieties, providing molecules with good film-forming abilities. High performance of OLEDs was assigned to the judicious combination of an enhanced charge transport ability due to the presence of the diphenylphosphine oxide group, the formation of pyrene excimers in the solid state and the assistance of the TADF property. More precisely, a contribution of a TADF process to the overall EL emission of OLEDs is suggested by the presence within the emissive layer of both pyrene and pyrene excimers, resulting in the presence of close-lying singlet and triplet states for the two forms. Besides, if a blue emission of the pyrene excimer assisted by TADF is suggested by the authors, no clear evidence of TADF is provided.

To support the presence of a TADF effect in the devices, the authors tentatively assigned the existence of the delayed component of fluorescence by the presence of close-lying singlet and triplet states in both pyrene derivatives and excimers, favorable to a reverse intersystem crossing giving rise to a delayed fluorescence. Multilayered OLEDs fabricated with **PO-4**–**PO-9** showed interesting efficiencies, with EQEs ranging from 7.2 to 9.1%. The contribution of the diphenylphosphine oxide group to the electron mobilities of these emitters was clearly evidenced by fabricating OLEDs using **PO-4**–**PO-9** as electron-carriers. By comparing with a reference electron-transport material, i.e., Alq_3_, a two-fold enhancement of EQEs could be determined while using these materials as electron-transport layers, evidencing their higher electron mobilities compared to that of tris(8-hydroxyquinoline)aluminum Alq_3_. Best OLEDs were obtained with **PO-8**, EQE peaking at 9.1%.

### 8. Benzonitrile derivatives

**Figure 15:**
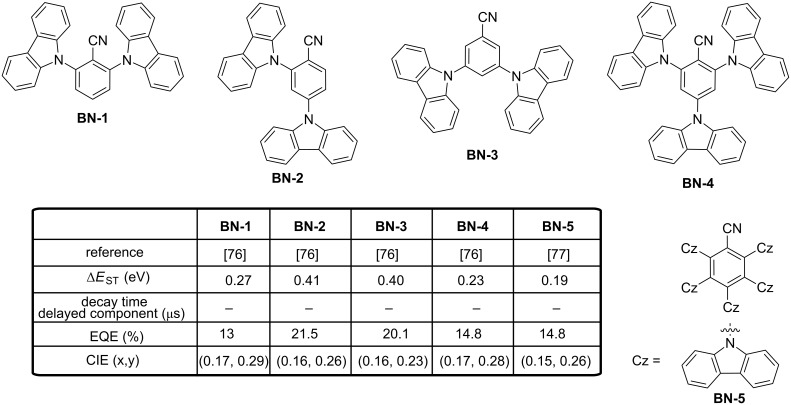
Benzonitrile-based emitters **BN-1**–**BN-5**.

In the search for new acceptors, benzonitrile was identified as a promising candidate capable to contribute to the design of deep blue TADF emitters. Precisely, the cyano moiety is a group limiting the size of electron acceptor moiety by its compacity while remaining one of the strongest electron-accepting groups at disposal for chemists. By combining benzonitrile with two or three carbazole units, and due to the planarity of the two structures (carbazole, benzonitrile), a sufficient steric hindrance could be induced to provide the highly twisted structures **BN-1**–**BN-4** (see Figure 15) [76]. The four carbazolyl benzonitrile derivatives **BN-1**–**BN-4** were easily prepared in a one-step approach through aromatic nucleophilic substitution. Encouraging results were obtained with the four emitters while using high-triplet-energy hosts with favorable carrier injection/transporting abilities.

**Figure 16:**
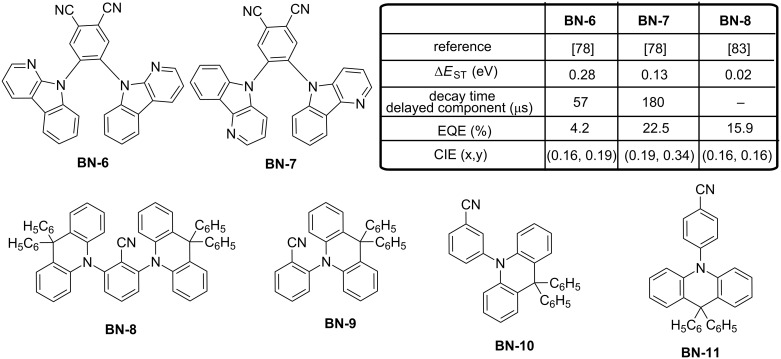
Benzonitrile-based emitters **BN-6**–**BN-11**.

The best performance was obtained with **BN-2**, endowing blue-emitting devices with a maximum EQE of 21.5%, which is among the highest values reported for blue TADF devices with an emission peak located at 470 nm. Another possibility could be to increase the number of carbazole units around the benzonitrile moiety. A benzonitrile derivative substituted by five carbazoles (**BN-5**) was synthesized and characterized by the Adachi team [77]. The OLEDs displayed a light-blue emission and a maximum EQE of 14.8%. Still based on this approach, the group of Hyuk Kwon went even further by introducing a nitrogen atom in the donor, furnishing the carbazole-derived α- and δ-carboline where the nitrogen heteroatom is introduced at the α- and δ-position respective to the central nitrogen atom (**BN-6** and **BN-7**, respectively, see Figure 16) [78]. Incorporation of carbolines in these two structures is justified by the fact that this group has recently been identified as an electron-transport material exhibiting a high triplet energy [79–82]. Even if the introduction of heteroatoms in aromatic compounds can increase the molecular relaxation, the bandgap and the triplet energies will simultaneously increase, consequently diminishing Δ*E*_ST_. Effectiveness of the strategy was clearly evidenced by the blue emission produced by OLEDs containing **BN-2** as the emitter (CIE coordinates of (0.19, 034), EL at 486 nm) and the high EQE of 22.5% attested of the TADF characteristics of the emitter. In contrast, **BN-1**-based devices demonstrated a low EQE of 4.2% resulting from its low PLQY (37% contrarily to 93% for **BN-2**) and the poor contribution of the delayed component to the overall emission (7% contrarily to 45% for **BN-2**). As a positive point, the EL spectrum of **BN-1**-based devices was blue shifted at 473 nm. Therefore, undeniably, it can be concluded that the effect of the heteroatom position in the carboline donor moiety is essential. Notably, for the two materials, the HOMO and LUMO energy levels of **BN-1** and **BN-2** are isolated from each other, but a partial overlap exists in **BN-1** due to the weaker donating ability of the α-carboline moiety. Jointly, theoretical calculations evidenced a larger bond length change between the ground and excited states for **BN-1** (0.048 Å vs 0.041 Å for **BN-2** between the carboline and the phenyl group). As a result of this, the higher molecular relaxation in **BN-1** is expected to favour the non-radiative processes, adversely affecting the EL performance. Another study revealed the importance of the donor moiety position compared to benzonitrile for high EL efficiency. In an effort to maximize the TADF process, Adachi developed a series of four highly twisted molecules **BN-8**–**BN11** consisting of the combination of 9,9-diphenylacridane donor unit(s) connected to a benzonitrile central core (see Figure 16) [83]. As first conclusions extracted from the theoretical calculations, the predicted Δ*E*_ST_ values were similar for all molecules (0.03 eV), suggesting that the substitution position has no effect on the up-conversion properties. Parallel to this, examination of the PL spectra of **BN-8**–**BN-11** showed the PL maximum to be located at 454 and 441 nm for **BN-8** and **BN-9**, respectively, whereas the emission was detected at 433 and 428 nm for the *meta*-substituted **BN-10** and *para*-substituted **BN-11**, respectively.

It was thus concluded that the π-conjugation was maximized upon *ortho*-substitution and the introduction of two donor units on **BN-8** optimized the delayed emission intensity so that **BN-8** was the only one to be tested in devices. OLEDs fabricated using **BN-8** as an emitter showed a blue emission at 463 nm (with CIE coordinates of (0.16, 0.16)) that coincides the PL emission maximum together with the high EQE of 15.9%. However, examination of the chemical stability of an encapsulated film of **BN-8** evidenced the emission intensity of the film to decrease in less than 5 min upon photoexcitation. Theoretical calculations pointed out the *ortho*-substitution to enhance the TADF efficiency because of the optimized steric hindrance but also to decrease the bond dissociation energy as a value of only 0.94 eV for the C–N bond was determined, much lower than the singlet and triplet energies of the molecules (2.75 eV and 2.73 eV, respectively).

### 9. Benzoylpyridine and di(pyridinyl)methanone–carbazole derivatives

**Figure 17:**
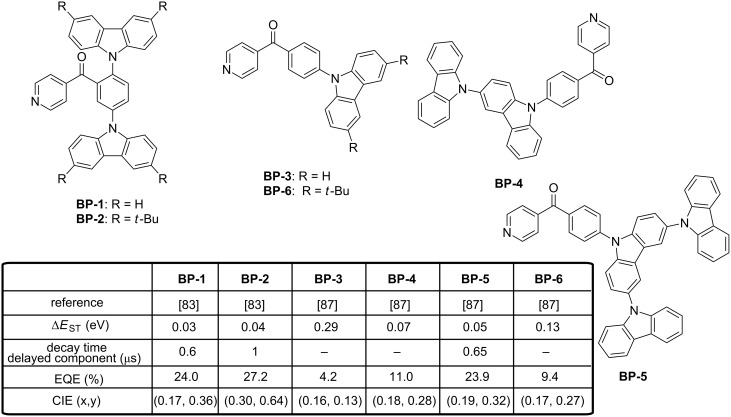
Benzoylpyridine-carbazole hybrid emitters **BP-1**–**BP-6**.

Emitters displaying efficient RISC and high PLQY are promising candidates for OLEDs and molecules comprising phenyl(pyridin-4-yl)methanone as the acceptor moiety are one of those. As first approach, the two carbazole donors were introduced at the *ortho-* and *meta*-positions of the phenyl ring of the acceptor (see Figure 17, **BP-1** and **BP-2**) [84]. Very small Δ*E*_ST_ of 0.03 and 0.04 eV and very high PL efficiencies of 88.0 and 91.4% were, respectively, determined for **BP-1** and **BP-2** in codoped films. These values are higher than that determined in solution for the two molecules (4.4 to 14.2% depending of the solvent for **BP-1**, 2.8 to 34.0% depending of the solvent for **BP-2**), demonstrating the suppression of the collisional and the intramolecular rotational quenching in thin films. However, the substitution pattern of carbazole drastically modified the emission wavelengths and a red-shift of approximately 20 nm was observed upon introduction of *tert*-butyl substituents on **BP-2**. Conversely, a higher electrochemical stability was determined for **BP-2** upon repeating CV scans, the two reactive C_3_ and C_6_ sites in *para*-position relative to the nitrogen atom of the carbazole being blocked by the *tert*-butyl groups. In multilayered devices, the bluer emitter **BP-1** provided efficiencies comparable to those obtained with iridium-based phosphorescent OLEDs at similar EL wavelength [85–86]. Notably, sky-blue **BP-1**-based OLEDs reached a maximum efficiency of 24% for the light peaking at 488 nm. The same year (2016), the same authors changed their strategy and combined all electron donors together, replacing the former D–A–D triads by D–A diads [87]. To tune the electron donating ability, carbazoles were introduced at the outer position of a carbazole unit, at the 3 and 3,6-conjugated positions of the first carbazole, resulting in donors composed in total of one to three carbazole groups. Comparison established with this series of emitters evidenced a clear decrease of Δ*E*_ST_ upon expending the size of the donating part and the number of carbazole units per donor. Thus, Δ*E*_ST_ decreased from 0.29 eV for **BP-3** to 0.07 eV for **BP-4** and 0.05 eV for **BP-5**, consistent with a higher spatial HOMO and LUMO separation and a more extended molecular HOMO orbital distribution.

**Figure 18:**
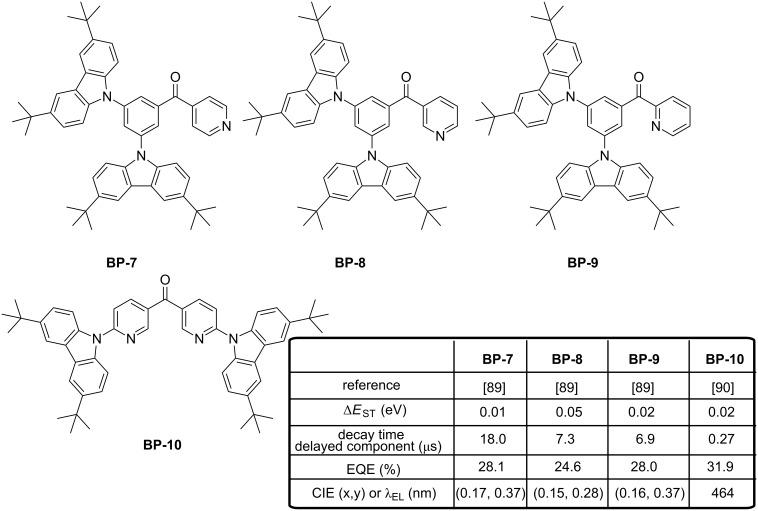
Benzoylpyridine-carbazole hybrid emitters **BP-7**–**BP-10**.

Unfortunately, despites these favorable features, a significant red-shift of the emission was evidenced for **BP-4** and **BP-5** as a result of a dual emission, one corresponding to a carbazole-centered π–π* transition at high energy and an additional but unexpected intramolecular charge transfer only observed for **BP-4** and **BP-5** at lower energy. A clear shift of the emission maximum was notably evidenced in toluene, the maximum emission wavelength shifting from 440 nm for **BP-3** to 480 nm for **BP-4** and 482 nm for **BP-5**. Therefore, only blue devices could be fabricated with the mono-substituted emitter **BP-3** and a comparison was established with **BP-6** differing from **BP-3** by the substitution pattern of the unique carbazole. Once again, a red-shift of the emission was observed upon incorporation of *tert*-butyl groups on carbazole, the emission in toluene being detected at 467 nm. Evaluation of the potential of **BP-3** and **BP-6** as new developed emitters for OLEDs confirmed the trend observed by PL and **BP-3** furnished a more blue OLED than **BP-6**, with an external efficiency peaking at 9.4%. By optimizing the device structure [88], the same authors could drastically increase the EQE of **BP-3**-based devices up to 18.4%, even if a non-negligible red-shift of the emission wavelength could be observed: 474 nm, (0.16, 0.25) for this study [88] contrarily to the previous emission detected at 452 nm, (0.13, 0.16) [87]. Inspired by the structure of **BP-2**, the same authors developed a series of three fluorescent molecules by varying the position of the nitrogen atom of the pyridine moieties **BP-7**–**BP-9** [89]. All molecules are characterized by high PLQYs in thin films, ranging from 92 to 97%, and small Δ*E*_ST_ varying from 0.01 eV for **BP-7** to 0.05 eV for **BP-8** and 0.02 for **BP-9**. Despites these appealing photophysical characteristics, positions of EL peaks appeared at 490, 476 and 490 nm for **BP-7**–**BP-9**-based devices, respectively, therefore in the blue-green region. While comparing with the standard triplet emitter Firpic, a clear enhancement of the EL performance was observed, EQE of Firpic-based OLEDs peaking at 18.7% whereas EQEs of 2.1, 24.6 and 28.0% could be, respectively, realized with the three TADF emitters **BP-7**–**BP-9** (see Figure 18). Here again, the ability of TADF emitters to outperform the standard phosphorescent emitters was demonstrated. Finally, the key to produce a pure blue emission with pyridine-based emitters seems to have been found with the di(pyridinyl)methanone electron-accepting core that could furnish a superior pure blue emission compared to emitters based on the benzoylpyridine core [90]. By introducing two pyridines in bis(6-(3,6-di-*tert*-butyl-9*H*-carbazol-9-yl)pyridin-3-yl)methanone (**BP-10**), a nearly planar molecule could be obtained, favouring the horizontal molecular orientation of the molecule within the co-doped emissive layer. By this specific arrangement in the EML, a perfect stacking of the molecules parallel to the substrate was determined, providing an isotropic orientation of the transition dipole moment. Finally, OLEDs fabricated with **BP-10** with a classical device structure furnished a record-breaking EQE of almost 32% with a relatively low dopant concentration (7 wt %) and an emission located at 464 nm.

### 10. Triazole derivatives

**Figure 19:**
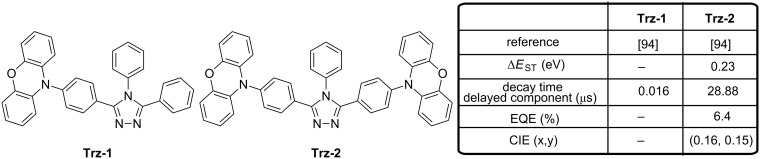
Triazole-based emitters **Trz-1** and **Trz-2**.

3,4,5-Triphenyl-4*H*-1,2,4-triazole is a good electron acceptor but also a remarkable electron-transport material used for the design of numerous OLED materials ranging from charge-transport materials to light-emitting materials [91–93]. Logically, combination of 3,4,5-triphenyl-4*H*-1,2,4-triazole with the electron-donor phenoxazine could provide emitters with TADF properties if conveniently associated and such assemblies were reported for the first time in 2013 (see Figure 19) [94]. Comparison of the diad **Trz-1** and the triad **Trz-2** evidenced in the absence of oxygen the triad **Trz-2** to be more luminescent than the diad **Trz-1** (29.8 and 43.1% for **Trz-1** and **Trz-2**, respectively). This trend was confirmed with the design of another series of diad/triads comprising an oxadiazole as the central electron acceptor. This characteristic is opposite to the trend classically reported in the literature where the molecules with a large oscillator strength show a high PLQY [95]. In the present case, the opposite situation was found as the more luminescent materials **Trz-2** showed the smaller oscillator strength, evidencing that the order of the PLQYs was not only controlled by the oscillator strength, but also by a competition with vibronic couplings responsible from nonradiative deactivation pathways. The fabrication of OLEDs with the most luminescent **Trz-2** furnished sky-blue OLEDs reflecting its PL spectrum in thin doped films (λ_EL_ = 456 nm, EQE = 6.4%).

### 11. Triphenylamine derivatives

**Figure 20:**
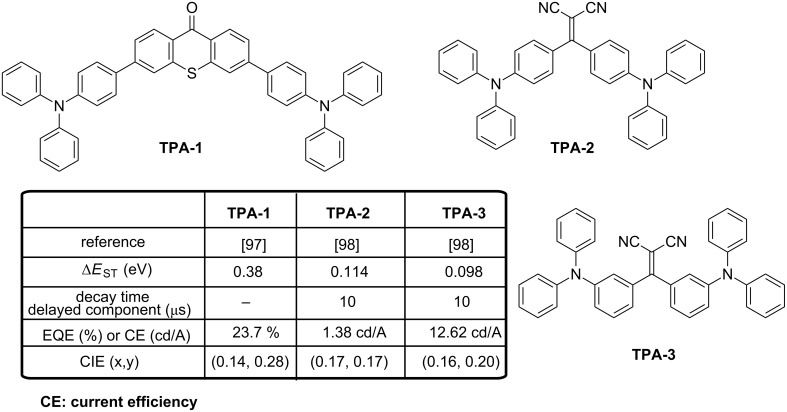
Triarylamine-based emitters **TPA-1**–**TPA-3**.

Triphenylamine is a remarkable electron-donating group that found applications in numerous research fields ranging from OLEDs to organic photovoltaics [96]. In the context of TADF blue emitters, an original strategy to tune the emission wavelength consisted in solely changing the sulfur atom valence state of the thioxanthone core, enabling the emission color to shift from blue to yellow [97]. Even if several connecting modes for the triphenylamine moieties onto the thioxanthone core was envisioned, a blue PL was only detected for **TPA-1** by introducing the two triphenylamine groups at the *para*-positions of the carbonyl group in 9*H*-thioxanthen-9-one (see Figure 20). Because of this specific substitution, a minimal HOMO/LUMO overlap was evidenced by theoretical calculations. Despites the symmetrical substitution of **TPA-1** and the reduction of the oscillator strength in the triad, the PLQY remained high, reaching 35% regardless doped or neat films under air conditions. In a standard device stacking, highly efficient emission could be realized as a maximum EQE value of 23.7% was obtained for OLEDs comprising an emissive layer with a doping concentration of 1 wt % and CIE coordinates of (0.139, 0.280).

In 2017, more blue OLEDs were obtained by using malononitrile as the electron acceptor [98]. The molecular orientation of the emitting material is essential to optimize the EL characteristics and an increase of the external efficiency by up to 46% can be achieved if the molecules are perfectly aligned horizontally by giving rise to light-outcoupling effects [99–101]. In this work, **TPA-2** and **TPA-3** share a similar Δ*E*_ST_ and similar PL characteristics but major differences were found upon fabrication of OLEDs with these two materials. Notably, the current efficiency of OLEDs elaborated with **TPA-3** as dopant was approximately 9 times higher than that determined for **TPA-2**-based OLEDs (12.6 and 1.4 cd/A, respectively). To explain these differences, the perfect horizontal orientation of **TPA-3** in doped films contrarily to the weak crystallinity and random orientation of **TPA-2** resulted in an improvement of the light extraction for **TPA-3**-based devices, justifying the enhanced performance.

## Conclusion

**Figure 21:**
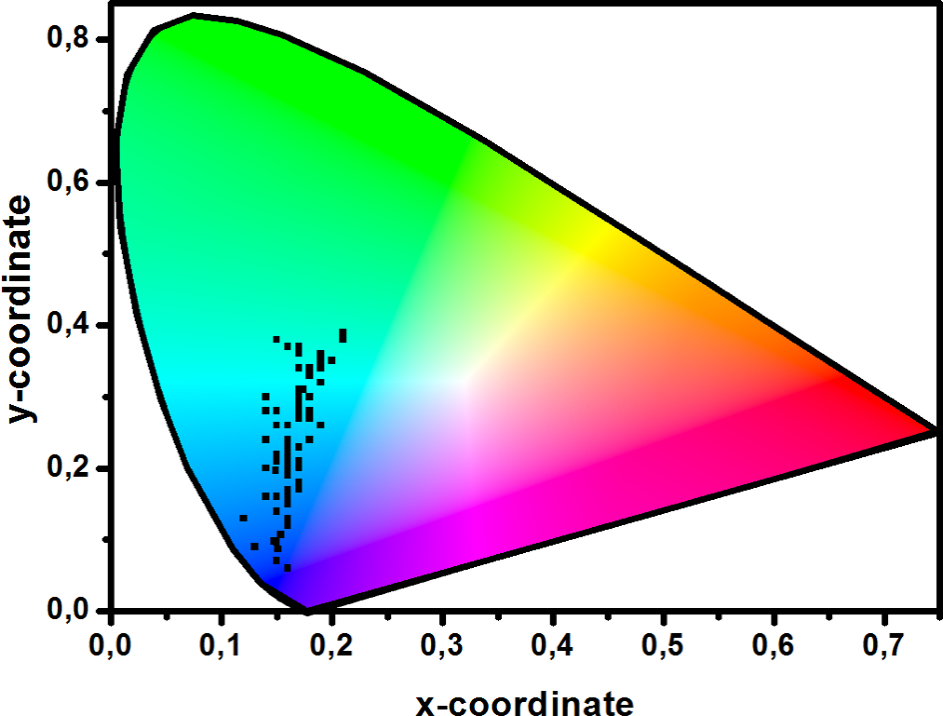
Distribution of the CIE coordinates of ca. 90 blue TADF emitters listed in this review.

To conclude, a wide range of strategies are currently developed to produce a blue TADF emission. Among the different findings that can constitute a guideline for the molecular design for blue TADF emitters, it can be cited: 1) The interruption of the π-conjugation by introducing an orthogonality between the donor and the acceptor to minimize the coupling between the two parts, 2) the fact to maintain the donor close to the acceptor to prevent a complete isolation of the donor and the acceptor, 3) the extension of the π-conjugated system of the donor and/or acceptor to maximize the oscillator strength and thus to increase the PLQY, 4) a minimization of ∆*E*_ST_ to optimize the rate constant of the reverse intersystem crossing, 5) the elaboration of light emitting materials with lifetimes of the delayed component of luminescence as short as possible to address the excited states annihilation issue, 6) a careful selection of the connectivity introduced between the electron donor/acceptor moieties as exemplified by the difference of the EL performance for materials differing by the substitution (*ortho-*, *meta-* and *para*-position of aromatic rings). The different results and observations reported in this review have clearly evidenced that a great deal of efforts has still to be done to produce a deep blue EL, as evidenced in Figure 21. At present, the bluest emitters reported in the literature, i.e., emitters with CIE *x*-coordinate below 0.16 and CIE *y*-coordinate below 0.10 only four are known: **D3** (0.15, 0.07) [29], reported in 2012, **T22** and **T23** (0.15, 0.10) [45], reported in 2017, and finally **CN-P8** (0.16, 0.06) [59], reported in 2016. **D3**, **T22** and **T23** are all based on carbazole, but carbazole is certainly not the best candidate for the design of highly stable deep blue emitter because of the photo-assisted electrochemical degradation processes it can initiate.

Since 2016, a great deal of efforts has been done to investigate new structures issued from communities other than Organic Electronics and electron donors such as phenoxaphosphine oxide or phenoxathiin dioxide and electron acceptors such as α- and δ-carbolines that have historically been used for the design of biologically active molecules are now commonly used during the elaboration of light emitting materials. Blue and stable emitters that will be developed in the future will certainly comprise such unprecedented moieties. Recently, another aspect of crucial importance to increase the EL performance concerns the molecular alignment of the emitter molecules in OLEDs as this can have an important effect on the outcoupling efficiency; this point warrants more systematic investigations in the future.
